# Atypical Phenotype of Myotonic Dystrophy Type 1 with Variant Repeats at the Age of Diagnosis

**DOI:** 10.3390/biology15131081

**Published:** 2026-07-06

**Authors:** Nemanja Radovanovic, Jovan Pesovic, Vanja Viric, Nikola Andrejic, Ivo Bozovic, Goran Brajuskovic, Dusanka Savic-Pavicevic, Stojan Peric

**Affiliations:** 1 Centre for Human Molecular, Faculty of Biology, University of Belgrade, Studentski Trg 16, 11000 Belgrade, Serbia; nemanja.radovanovic@bio.bg.ac.rs (N.R.); jovan.pesovic@bio.bg.ac.rs (J.P.); brajuskovic@bio.bg.ac.rs (G.B.); 2 Neurology Clinic, University Clinical Centre of Serbia, Dr Subotica Starijeg 6, 11000 Belgrade, Serbia; vanja.viric97@gmail.com (V.V.); ds245003@student.med.bg.ac.rs (N.A.); ivo.bozovic20@gmail.com (I.B.); 3 Faculty of Medicine, University of Belgrade, Dr Subotica Starijeg 6, 11000 Belgrade, Serbia

**Keywords:** myotonic dystrophy type 1, variant repeats, repeat interruptions, *DMPK*, repeat expansion, genetic modifier, age at diagnosis

## Abstract

Myotonic dystrophy type 1 (DM1) is caused by an expansion of CTG repeats in the *DMPK* gene. In a proportion of patients, the expanded allele contains variant repeats, which have been associated with later disease onset and different clinical presentation, although their full impact remains incompletely defined. We compared sociodemographic, neuromuscular, and multisystem clinical features between DM1 patients with pure CTG expansions (*n* = 66) and those with variant repeats (*n* = 9), who formed a consecutive cohort of unrelated index cases evaluated at the age of diagnosis in routine clinical practice. Patients with variant repeats were nine years older at diagnosis than patients with pure repeat expansions (*p* = 0.025), had more years of formal education (*p* = 0.024), and showed reduced muscle strength in proximal lower limbs (*p* = 0.049). No childhood or juvenile forms were observed among patients with variant repeats. Sex, disease duration, and most other clinical parameters, including multisystem involvement, did not differ between groups. The results of our exploratory study support variant repeats as disease modifiers in both age at onset and pattern of muscle involvement, and imply a two-sequential-component hypothesis in DM1 pathogenesis.

## Simple Summary

Myotonic dystrophy type 1 is an inherited disease that affects muscles and other parts of the body. It is caused by an abnormally long, repeated CTG sequence in the *DMPK* gene. In some patients, this sequence contains other kinds of repeats, called variant repeats. These changes may influence when the disease starts and how it appears, but their exact clinical effect is still not fully understood. In this study, we found that patients with variant repeats were usually diagnosed later in life, had spent more years in school, and had weaker muscles of the hips and thighs compared to patients without variant repeats. However, most other health problems related to the disease were similar between the two groups. These results suggest that variant repeats may alter how the disease develops and appears. Understanding how variant repeats modify multisystemic appearance of myotonic dystrophy type 1 may help clinicians recognize unusual presentations and improve patient selection for clinical trials.

## 1. Introduction

Myotonic dystrophy type 1 (DM1) is the most common muscular dystrophy in adults, with an estimated clinical prevalence of approximately 1 in 8000 [1], although more recent population genetic screening has suggested the true prevalence may be considerably higher, estimated at approximately 1 in 2100 [2]. DM1 is caused by an expansion of CTG repeats in the 3′ untranslated region of the *DMPK* gene [3,4,5]. It is a progressive multisystem disorder affecting skeletal muscle, heart, central nervous system, eyes, and endocrine system, among others [1,6]. The clinical spectrum ranges from congenital to late-onset forms, and DM1 is considered one of the most variable human disorders [1,7]. The main pathogenic mechanism is an RNA-mediated toxic gain of function, in which expanded CUG transcripts sequester RNA-binding proteins and disrupt alternative splicing [8,9]. Although CTG repeat length broadly correlates with disease severity and age at onset, it does not fully account for the observed phenotypic variability [10,11]. The expanded repeat is unstable in the germline, with a bias toward further expansion during intergenerational transmission, which underlies genetic anticipation [12]. The repeats are also somatically unstable and this instability has been shown to modify age at disease onset [11,13]. In addition, we and others have recognized variant repeats (or repeat interruptions) within the CTG tract as modifiers of the DM1 phenotype [14].

Variant repeats within the CTG tract are present in a subset of DM1 patients, with estimated frequencies ranging from 3% to 11% across different cohorts [15,16,17,18,19,20,21,22,23]. The most common repeat interruption is CCG, although CTC, GGC, and CAG motifs have also been reported. Variant repeats have been associated with reduced somatic instability and more stable intergenerational transmission compared to pure CTG expansions [16,18,19,23,24,25]. To date, no congenital or childhood DM1 cases with variant repeats have been reported [14]. The clinical presentation among patients with variant repeats has shown to be diverse across studies. Most reports describe milder or atypical phenotypes with a later disease onset than expected, often with less severe manifestations in certain clinical domains, particularly cognitive function, and in some cases with atypical patterns of muscle involvement, including proximal or axial muscle weakness [17,19,20,21,22,26]. On the other hand, some studies observed no major differences between patients with and without interruptions apart from cognitive involvement [17], and even a severe phenotype despite late disease onset [26]. Notably, previous studies have primarily identified patients with variant repeats through retrospective screening of existing DM1 cohorts and including relatives of previously diagnosed patients. Here, we conducted an exploratory study aiming to compare sociodemographic, neuromuscular, and multisystem clinical features between DM1 patients carrying pure and interrupted CTG expansions in a consecutive cohort of unrelated index cases evaluated at the age of diagnosis in routine clinical practice.

## 2. Materials and Methods

### 2.1. Study Design and Participants

This retrospective observational study included 75 consecutive, unrelated index cases at the time of diagnosis and entry in the Registry for myotonic dystrophies. Patients were examined at the Outpatient and Inpatient Units of the Neurology Clinic, University Clinical Centre of Serbia, Belgrade, between November 2017 and December 2025. Patients diagnosed through family screening and those with congenital DM1 were excluded. All patients underwent standard neurological examination and peripheral blood sampling for genetic testing. The diagnosis of DM1 was established based on characteristic clinical features and electromyography findings, and was confirmed by molecular genetic analysis at the Centre for Human Molecular Genetics, University of Belgrade–Faculty of Biology. The establishment of the Serbian registry for rare neuromuscular diseases, including myotonic dystrophies, was approved by the Ethics Committee of the University of Belgrade–Faculty of Medicine (decision 29/XI-7 from 20 November 2013) and by the Ethics Committee of the University Clinical Centre of Serbia (decision 18/10 from 23 February 2026). These approvals permit the publication of anonymous patient data in scientific publications without additional approval from the Ethics Committee and without the need for additional informed consent from patients. The study was conducted in accordance with the Declaration of Helsinki.

### 2.2. Genetic Analysis

Genomic DNA was extracted from peripheral blood using the QIAamp^®^ DNA Blood Mini Kit (Qiagen, Hilden, Germany). The diagnosis of DM1 was confirmed by repeat-primed polymerase chain reaction (RP-PCR) targeting expanded CTG repeats in the *DMPK* gene from both the 5′ and 3′ ends [27]. This approach also enabled screening for repeat interruptions within the ends of expanded alleles. Capillary electrophoresis was performed on ABI3130 and ABI3500 Genetic Analyzers (Applied Biosystems, Thermo Fisher Scientific, Waltham, MA, USA) using GeneScan™ 500 LIZ^®^ and GeneScan™ 600 LIZ^®^ as internal size standards. Samples showing atypical or discontinuous peak patterns suggestive of repeat interruptions were further analyzed using variant-specific primers to confirm the presence of interruptions, as previously described [19]. Myotonic dystrophy type 2 (DM2) was excluded in all patients by RP-PCR targeting repeat expansion in the *CNBP* gene [27]. Patients were stratified according to repeat expansion structure into those carrying pure CTG repeat expansions and those carrying variant repeats.

### 2.3. Clinical Assessment

Detailed sociodemographic and clinical data were collected for all patients at the time of diagnosis. Sociodemographic variables included sex and educational level, expressed as years of formal education, which served as an indirect proxy of cognitive function. General disease characteristics included age at diagnosis, age at disease onset, and disease duration. Age at disease onset was defined as the age at which the first muscular symptoms, including muscle weakness or myotonia, were recognized by the patient or their family members.

Disease severity was assessed using the Muscular Impairment Rating Scale (MIRS) [28], a five-point ordinal scale specific to DM1 that reflects the characteristic distal-to-proximal progression of muscle weakness. For statistical analyses, MIRS scores were analyzed both as an ordinal variable and as a binary variable, with grades 1–3 classified as mild and grades 4–5 as severe muscular impairment. Muscle strength was evaluated by two neurologists (S.P. and I.B.) using manual muscle testing according to the Medical Research Council (MRC) 0–5 scale [29], where 5 indicates normal muscle strength and 0 indicates no muscle contraction. The following muscle groups were examined: shoulder abductors and adductors and elbow flexors and extensors for proximal upper extremities; wrist and finger flexors and extensors, finger abductors and adductors for distal upper extremities; hip flexors, extensors, abductors and adductors and knee flexors and extensors for proximal lower extremities; and ankle and toe plantar and dorsiflexors for distal lower extremities. For each anatomical region, the weakest muscle score was recorded, and the scores from all regions were summed to obtain the total MRC score. A maximum possible score of 20 indicated preserved muscle strength across all tested muscle groups [30]. Additionally recorded neuromuscular manifestations included ptosis, facial weakness, nasal speech, bulbar symptoms (dysarthria and dysphagia), hand locking as reported by patients, grip and percussion myotonia, myotonia of the masseter and tongue, gait difficulty, and ambulation impairment (defined as the use of any walking aid or wheelchair). Electrophysiological findings included electromyography evidence of myotonia and myopathy, as well as electroneurography evidence of polyneuropathy.

Systemic evaluation encompassed multiple organ domains. Respiratory assessment included spirometry measurement of forced vital capacity (FVC) (with FVC < 80% considered indicative of pulmonary restriction), as well as the presence or absence of respiratory support (non-invasive ventilation). Cardiac evaluation included electrocardiographic (ECG) findings, documentation of pacemaker implantation, and patient-reported cardiac symptoms. Severe ECG abnormalities were defined according to Groh et al. as the presence of at least one of the following: non-sinus rhythm, PR interval ≥ 240 ms, QRS duration ≥ 120 ms, or second- or third-degree atrioventricular (AV) block [31]. Skeletal assessment included the presence of high-arched palate, scoliosis, kyphosis, and lumbar lordosis. Endocrine evaluation included assessment of infertility and glucose metabolism abnormalities. Glucose metabolism abnormalities were analyzed as a binary variable and included documented prediabetes, diabetes mellitus, or use of antidiabetic therapy.

Laboratory and metabolic parameters included serum creatine kinase (CK), total cholesterol, and triglycerides levels. Serum CK was analyzed as a continuous variable. Total cholesterol and triglycerides were analyzed as binary variables (normal vs. above the upper limit of normal), with classifications based on standard laboratory reference ranges from the local laboratory.

### 2.4. Statistical Analysis

Statistical analyses were performed to compare sociodemographic and clinical characteristics between patients with pure CTG repeat expansions and those carrying variant repeats. Normality of continuous variables was assessed using the Shapiro–Wilk test. Group comparisons were performed using the Welch t-test for normally distributed continuous variables, the Mann–Whitney U test for non-normally distributed continuous or ordinal variables, and Fisher’s exact test for categorical variables. All tests were two-sided, and *p* < 0.05 was considered statistically significant. All analyses were performed using available data for each variable, resulting in variable sample sizes across parameters. Given the exploratory nature of the study and the small number of patients with variant repeats, no formal correction for multiple testing was applied. Therefore, *p*-values should be interpreted as nominal. Effect sizes were calculated for parameters showing nominal statistical significance (*p* < 0.05) or borderline significance (0.05 < *p* < 0.1) in order to interpret the magnitude of group differences [32]. Hedges’ g was used for normally distributed continuous variables, and Cliff’s delta was used for ordinal or non-parametric comparisons. Statistical analyses were performed in R version 4.5.1 (R Foundation for Statistical Computing, Vienna, Austria) [33].

## 3. Results

### 3.1. Sociodemographic and General Disease Characteristics

**Figure 1:**
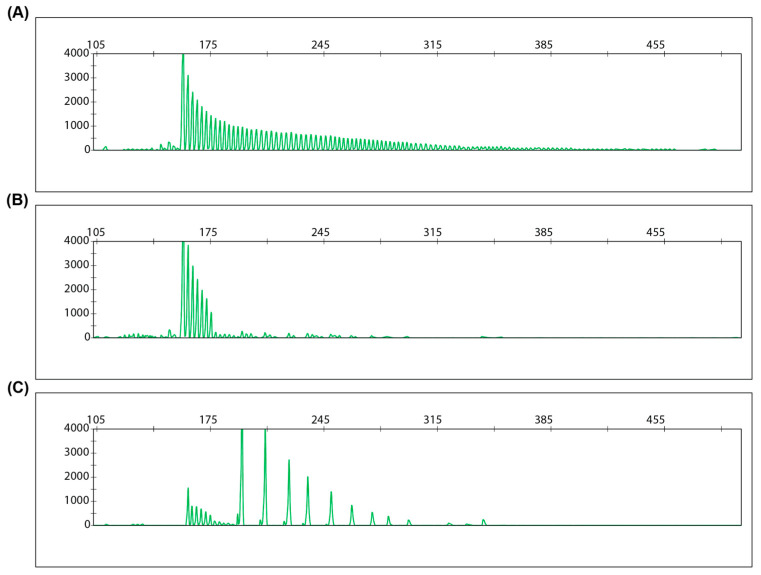
Representative repeat-primed PCR electropherograms of pure and interrupted *DMPK* CTG expansions. (**A**) A patient with a pure CTG expansion showing the characteristic continuous ladder pattern with gradually decreasing peak heights; (**B**) A patient with variant repeats showing a discontinuous ladder pattern with gaps between peaks, suggestive of repeat interruptions; (**C**) The same patient shown in (**B**) analyzed using CCG-specific primers, confirming the presence of CCG variant repeats at the 3′ end of the expanded allele. The x-axis represents fragment size in nucleotides, and the y-axis represents fluorescence intensity in relative fluorescence units.

Among the 75 consecutive index cases with DM1 included in the study, 66 carried pure CTG repeat expansions and nine carried variant repeats (12.0%). In all nine patients CCG variant repeats were identified, located at the 3′ end of the CTG expansion in eight patients and at the 5′ end in one patient (Figure 1).

Sociodemographic and general disease characteristics are summarized in Table 1. Sex distribution and disease duration did not differ between groups (*p* > 0.05). Age at diagnosis was significantly higher in the variant repeat group with an average difference of 8.7 years and a moderate-to-large effect size (49.0 ± 9.2 vs. 40.3 ± 11.6 years; Welch’s t-test, *p* = 0.025; Hedges’ g = 0.75). Similarly, age at disease onset was on average 8.5 years later in patients with variant repeats compared with those carrying pure CTG expansions (37.0 ± 13.0 vs. 28.4 ± 13.6 years), although this difference did not reach statistical significance (Welch’s t-test, *p* = 0.116). The distribution of disease forms differed significantly between groups (Fisher’s exact test, *p* = 0.023). Patients with variant repeats presented only with adult and late-onset forms (9/9, 100%), whereas childhood and juvenile forms were observed only in patients with pure repeats (25/63, 39.7%), along with adult and late-onset forms. Educational level was significantly higher in patients with variant repeats, with a large effect size (13.3 ± 2.0 vs. 11.1 ± 2.2 years; Welch’s t-test, *p* = 0.024; Hedges’ g = 1.00).

### 3.2. Neuromuscular Characteristics

Neuromuscular involvement and muscle strength measures are summarized in Table 2. Overall disease severity, assessed using MIRS, did not differ significantly between patients with pure repeats and those carrying variant repeats (*p* > 0.05). Total muscle strength, quantified by the summed MRC score, was also comparable between groups (16.18 ± 1.84 for pure repeats and 15.63 ± 2.83 for variant repeats; Mann–Whitney U test, *p* = 0.582). Regarding regional muscle strength measures, a significant difference was observed for proximal lower-extremity muscle strength, with lower scores in patients carrying variant repeats compared with those carrying pure repeats, with a moderate effect size (4.25 ± 0.71 vs. 4.69 ± 0.50; Mann–Whitney U test, *p* = 0.049; Cliff’s delta = −0.36). No significant differences were observed for proximal or distal upper extremity strength or for distal lower extremity strength (*p* > 0.05).

Among other neuromuscular variables, nasal speech was less frequent in patients with variant repeats than in those with pure repeats (50.0% vs. 79.7%), showing a trend toward statistical significance (Fisher’s exact test, *p* = 0.083). Other neuromuscular features, including other bulbar symptoms, myotonia, and muscle involvement across examined muscle groups, did not differ between groups (*p* > 0.05).

### 3.3. Multisystem Involvement

Multisystem clinical features are summarized in Table 3. No significant differences were observed between groups across multisystem parameters, including skeletal, electrophysiological, respiratory, cardiac, ophthalmological, and endocrine parameters (*p* > 0.05).

### 3.4. Metabolic and Laboratory Parameters

Metabolic and laboratory parameters are summarized in Table 4. Serum CK levels were higher in patients with variant repeats than in those carrying pure repeats (326.7 ± 157.0 vs. 227.2 ± 164.2 U/L), showing a trend toward statistical significance and a moderate-to-large effect size (Mann–Whitney U test, *p* = 0.071; Cliff’s delta = 0.44). No significant differences were observed between groups in the proportions of patients with elevated total cholesterol or triglycerides (*p* > 0.05).

## 4. Discussion

In this exploratory study, we found that DM1 patients carrying variant repeats showed different phenotype at the age of diagnosis in comparison to those without variant repeats. Specifically, patients with variant repeats were older at diagnosis, had higher formal education as a proxy of cognitive function, and, notably, displayed a different pattern of muscle involvement, with more pronounced weakness of the proximal lower extremity. At the same time, both patient groups did not differ significantly in terms of disease duration or other examined clinical parameters. Taken together, our results highlight that differences in age, cognitive proxy, and pattern of muscle involvement are present even at the age of diagnosis, while other clinical parameters show a convergence toward a typical DM1 phenotype.

The proportion of patients with variant repeats in our cohort was 12.0% (9/75), which is higher than usually reported. Previous studies, including ours, have most consistently reported frequencies between 3% and 5% [15,16,17,18,19]. However, higher frequencies have also been reported in larger cohorts, including 8.9% (17/191 expanded alleles) in the Saguenay-Lac-Saint-Jean cohort [23] and 8.4% (21/250 patients) in the OPTIMISTIC cohort, with site-specific frequencies ranging from 4.5% in Paris, France, to 13.6% in Nijmegen, The Netherlands [20]. Our figure therefore lies at the higher end of the reported range. This may reflect differences in how patients were identified, given that our cohort consisted of consecutive, unrelated index cases diagnosed in routine clinical practice. It was suggested that the higher frequency of variant repeat carriers in the OPTIMISTIC trial may be due to over-recruitment of patients with preserved cognition and higher motivation to participate in a clinical trial [20]. A similar bias may be present in this study, as patients with better formal education may be more likely to seek medical attention, and subsequently be included in the clinical registry.

Patients with variant repeats in our cohort presented with disease symptoms later in life. The age at diagnosis was approximately nine years later in patients with variant repeats compared to those with pure repeats, and the age at onset was similarly delayed by around nine years. At the same time, disease duration was nearly identical between groups (around 12 years). This finding indicates that the later age at diagnosis was not explained by longer disease duration (i.e., slower progression) but solely by a later disease onset. Consistent delays in age at onset among patients with variant repeats have been reported across multiple cohorts independently of the study design. Miller et al. [21] and Wenninger et al. [22] found that the mean age at onset was around seven years later in patients with repeat interruptions (32 vs. 24 years). Cumming et al. [20] reported that patients with variant repeats in the OPTIMISTIC cohort had an age at onset on average 13 years later than predicted by the best estimate of progenitor expansion length alone. Therefore, the later presentation observed in our cohort is consistent with the literature. On the other hand, the difference in this study is particularly robust at the level of age at diagnosis, which is considered a more objective measure than recalled onset and was observable due to our study design. It is important to note that age at onset is inherently subjective and may be influenced by cognitive and behavioral involvement in DM1, which can impair symptom awareness and recall [34]. Since patients with variant repeats have been reported to have less cognitive impairment [17,19,21]; they may recall initial symptoms more accurately. Conversely, patients with pure repeats may have greater cognitive involvement and reduced awareness of early symptoms. The magnitude of any resulting bias cannot be determined from the available data. Furthermore, no congenital, childhood, or juvenile forms were observed among patients with variant repeats in this cohort; i.e., all patients with interruptions presented with either adult or late-onset disease form. To our knowledge, no congenital or childhood DM1 cases with variant repeats have been reported to date [14]. In contrast, juvenile-onset cases have been reported in a small number of families with variant repeats. In our previous cohort, we [19] reported two patients with onset at ages 12 and 15 in families carrying interrupted expansions, while Braida et al. [16] and Botta et al. [18] each reported one patient with onset at age of 17 and 15, respectively. These cases indicate that juvenile onset can occur in carriers of interrupted expansions, although it is less common. Musova et al. [15] reported a patient with myotonia since age seven, but this case involved an interrupted intermediate-length allele (37 repeats), and the pathogenic contribution of that allele alone remains uncertain, with the possibility of a concomitant chloride channel mutation [14]. Overall, the available evidence supports a consistent shift toward later disease expression in patients with variant repeats, and consequently later diagnosis.

Patients with variant repeats in our cohort had, on average, two more years of formal education compared to those with pure expansion. Educational attainment can be used as a proxy of cognitive function. However, this measure should be interpreted with caution since it is influenced by factors beyond cognition, including socioeconomic background and access to education, and is not equivalent to standardized neuropsychological assessment. Nevertheless, this observation is consistent with previous studies. Santoro et al. [17] reported that cognitive involvement was the only domain in which variant and non-variant DM1 patients differed, with none of the variant repeat patients showing significant cognitive impairment at a mean age of 60 years. We previously reported [19] normal Addenbrooke’s Cognitive Examination Revised scores in all tested patients with variant repeats, while Miller et al. [21] showed that the variant-repeat group performed better across several cognitive and behavioral measures, including full scale IQ, processing speed, and depression scores. In addition, Cumming et al. [20] identified variant repeats, together with age at sampling and expansion size, as significant explanatory variables for better attention, executive function, social behavior and less apathy, fatigue, and daytime sleepiness in the OPTIMISTIC cohort. Collectively, these findings provide evidence that central nervous system involvement may be less pronounced in patients carrying interrupted expansions.

The notable neuromuscular finding in our cohort was weaker proximal lower-extremity strength in patients with variant repeats. Consistent with this, ambulation impairment was observed in 22.2% of patients with variant repeats compared with 4.7% of those with pure repeats, although this difference did not reach statistical significance. This observation is clinically relevant because proximal lower-extremity muscles are essential for standing, rising from a seated position, and walking. This pattern differs from the usual distal-predominant weakness observed in classic DM1 and aligns with our previous reports of atypical or DM2-like presentations in patients with interrupted expansions, where we described that four out of eight patients with variant repeats presented with proximal leg weakness, calf hypertrophy, and absent percussion myotonia, with even an initial clinical suspicion of DM2 [19]. Similarly, Santoro et al. [17] reported proximal muscle weakness in two out of five patients with variant repeats. Notably, Ballester-Lopez et al. [26] described DM1 patients with variant repeats from the same family with severe axial and proximal weakness, including dropped head and respiratory involvement, despite late disease onset. In a large Canadian cohort, Overend et al. [23] demonstrated that variant repeats significantly modified skeletal muscle strength across multiple muscles, including knee extensors. Patients with variant repeats showed better strength than predicted by their expansion size. While their findings support a protective effect of variant repeats on overall muscle strength, our results suggest that variant repeats may also involve a shift in the regional distribution of weakness, with relatively more prominent proximal lower-extremity involvement. Taken together, these findings suggest that repeat interruptions are associated with a modified pattern of muscle involvement rather than a globally milder phenotype. Additionally, the current study supports this by demonstrating that a modified pattern can be observed as early as the age at diagnosis. This highlights the need for parallel molecular testing for both DM1 and DM2, particularly in patients presenting with atypical muscle weakness patterns. Serum CK levels were somewhat higher in our patients with variant repeats, which further complicates differential diagnosis, since CK increase is usually observed in DM2 and not in DM1. We are not aware of consistent literature evidence linking CK elevation specifically to variant repeat status in DM1. For example, in the family reported by Ballester-López et al. [26], CK values were largely normal, whereas Cumming et al. [35] described a patient with a CCG-interrupted expansion and a CK of 468 IU/L. However, in the context of atypical symptoms in a patient with DM1, elevated CK may be a feature that prompts consideration of variant repeats. In addition, the presence of proximal muscle weakness at the age of diagnosis raises the question about the suitability of MIRS for assessing disease severity in DM1 patients with variant repeats, since five of eight patients in our study were graded as MIRS stage 4 or 5. MIRS is based on the typical distal-to-proximal progression of muscle weakness in DM1 and may be less suitable in patients with variant repeats, in whom proximal involvement may occur earlier and be relatively more prominent than expected for classical DM1. In such cases, MIRS may overestimate disease advancement relative to the patient’s true functional status.

We did not observe differences in multisystem involvement between groups at the age of diagnosis. The available literature does not support a mitigating effect of variant repeats across systems other than the central nervous system [17,19,21].

**Figure 2:**
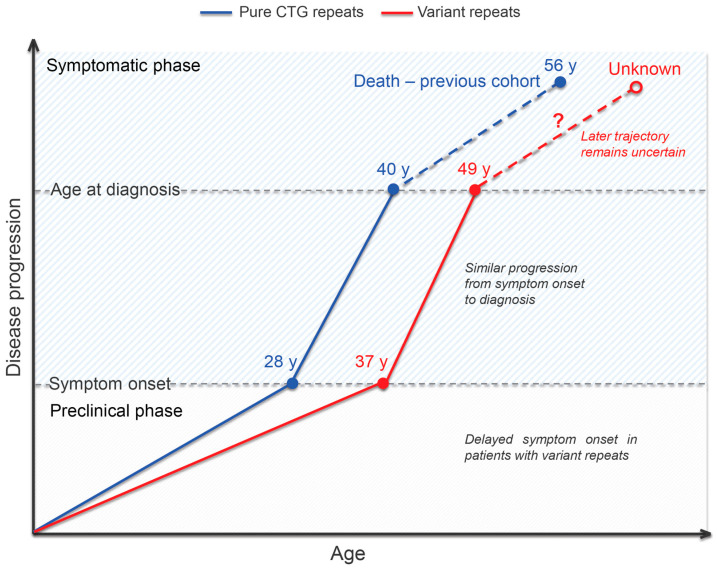
Schematic representation of disease progression in DM1 patients with pure CTG repeats and variant repeats. Patients with variant repeats showed later symptom onset and later age at diagnosis, while the time interval from symptom onset to diagnosis was similar between groups. The dashed red line indicates that the later disease trajectory of patients with variant repeats remains uncertain. The age at death shown for the pure-repeat group is based on a previous Serbian DM1 cohort and is included only as contextual information, not as an outcome of the present cohort. Ages are shown as rounded group mean values. The figure is schematic and not drawn to scale.

Findings from previous studies and from our study enabled us to compare disease progression in DM1 patients with and without variant repeats (Figure 2). Patients with variant repeats have a delayed age at onset [19,20,21,22], but once symptoms appear, the rate of disease progression appears to be similar in both groups, as indicated by nearly identical time intervals between age at onset and age at diagnosis. In addition, the disease progression in patients with variant repeats shows some qualitative differences rather than a consistently milder course. These differences include more pronounced impairment of proximal lower-extremity muscles, more preserved cognitive function, and a convergence of other system involvement toward a typical DM1 phenotype, highlighting a tissue-specific modifying effect of variant repeats. It remains unclear whether the modifying effect of variant repeats persists after diagnosis. There is some indication that the apparent protective effect of interruptions becomes less apparent with age. For example, Santoro et al. [17] described a patient with only 65 repeats and interruptions who developed a full multisystemic phenotype 40 years after disease onset. Similarly, Ballester-López et al. [26] reported that patients with repeat interruptions, despite first symptoms occurring after age 50, developed severe multisystemic involvement after age 60. In addition, no systematic data on survival in DM1 patients with variant repeats are available. In a Serbian cohort of 171 DM1 patients, the mean age at death was approximately 56 years [36] (Figure 2), which is in line with reports from other DM1 cohorts, ranging from 53 to 59 years [37,38]. In studies of patients with variant repeats, the oldest patients reported alive were in their early 70s [18,26], and no deaths were reported. Among Serbian patients with variant repeats identified to date [19,25], including those from the current study, only one is known to have died, at the age of 75, due to acute ischemic stroke. Several others are in their 60s and 70s. However, these observations do not allow conclusions about survival, but are consistent with delayed disease expression in patients with variant repeats.

The molecular basis for the observed clinical differences in patients with variant repeats is thought to operate primarily through stabilization of the *DMPK* expansion. Several studies have shown that variant repeats are associated with stabilization or even contraction of the expanded allele during intergenerational transmission [16,18,19,24]. The absence of congenital cases likely reflects the stabilizing effect of repeat interruptions on intergenerational transmission, which reduces the likelihood of large expansions in offspring that characterize congenital and childhood-onset forms [16,18,19,24,25]. In addition, reduced somatic instability of *DMPK* expansions and its association with delayed age at onset [20,23,25] support the role of somatic instability as a modifier of DM1 [11]. Although interrupted expansions are more somatically stable, they remain biased toward further expansion over time [16,25], which may be consistent with systemic convergence toward a typical DM1 phenotype as the disease progresses. Santoro et al. [17] also showed that interrupted alleles still produce ribonuclear foci and splicing abnormalities similar to those observed in typical DM1 muscle, suggesting that the core RNA-mediated pathogenic mechanism is preserved in the presence of repeat interruptions.

The association between delayed age at onset and reduced somatic instability in DM1 patients with variant repeats [16,24,25] has enhanced the role of somatic instability in DM1 molecular pathogenesis [11,13]. Our findings suggest an additional contribution of instability to DM1 pathogenesis. Observation of later diagnosis in patients with variant repeats despite similar disease duration from the onset of initial symptoms (Figure 2) and the absence of significant differences in most multisystem symptoms between patients with and without repeat interruptions imply that DM1 pathogenesis may involve two sequential, mechanistically distinct components, as demonstrated in Huntington’s disease [39,40]. According to this model, inherited expansions tend to undergo further somatic expansion, and upon reaching a critical, cell type-specific pathogenic threshold, downstream mechanisms that induce cell damage, dysfunction and loss are activated. Since variant repeats stabilize *DMPK* expansions in somatic cells, reaching the critical threshold is delayed, explaining later age at onset. Once the interrupted expansion exceeds the pathogenic threshold, core RNA-mediated toxicity and other downstream mechanisms are triggered, driving symptom progression at a rate similar to pure expansions, and resulting in comparable multisystem involvement at diagnosis in both patient groups. This is in line with evidence on a transgenic DM1 mouse model that long-term exposure to toxic RNA contributes to symptom progression even when somatic instability is controlled [41]. Qualitative differences in disease symptoms, such as preserved cognitive function and atypical proximal weakness, may arise from differential somatic expansion rates across cell types and from cell type-specific effects of variant repeats on the structure and protein-binding properties of toxic RNA. The two-sequential-component hypothesis in DM1 pathogenesis deserves further investigation.

The main limitation of our study is the small number of patients with variant repeats. This reflects the low frequency of interrupted expansions, but it also limits statistical power, increasing the possibility that some clinically relevant differences have remained undetected. Multiple clinical comparisons were performed without formal correction for multiple testing. Accordingly, nominally significant findings should be interpreted with caution and require confirmation in larger cohorts. Another limitation is that cognitive function was assessed indirectly using years of formal education rather than standardized neuropsychological testing. Unfortunately, strength of individual muscles was not retained as a separate variable, and therefore we were not able to compare individual muscle strength between the two groups. We believe that such comparisons may better define pattern of muscle involvement in DM1 patients with and without variant repeats and are planned as part of our future research. The cross-sectional study design further limits the ability to draw conclusions regarding disease progression over time. Larger cohorts and longitudinal studies are required to define more precisely which symptoms are consistently modified by interrupted expansions and at what disease stage these differences become less apparent compared to patients with pure repeats.

## 5. Conclusions

In conclusion, our exploratory study revealed that interruptions in DM1 were associated with a later age at diagnosis, better education, and an atypical pattern of muscle involvement with proximal lower-extremity weakness. These findings support the role of variant repeats as modifiers of disease expression. At the same time, the majority of clinical parameters did not differ significantly between groups. This pattern may reflect phenotypic convergence as disease advances, during which the initial modifying effect of interruptions progressively attenuates. This interpretation carries practical implications that patients with variant repeats should not be presumed to follow a consistently milder disease course, particularly with aging. Screening for variant repeats should be considered for all DM1 patients, particularly those with atypical presentations that may resemble DM2, and in the context of clinical trial design, where variant repeat carriers may represent statistical outliers.

## Figures and Tables

**Table 1:** Sociodemographic and general disease characteristics of consecutive patients with myotonic dystrophy type 1 with pure and variant repeats at the age at diagnosis.

Variable	Pure Repeats	Variant Repeats	*p*-Value ^1^
Number of patients	66	9	
Sex (female)	35 (53.0%)	5 (55.6%)	1.000 ^2^
Age at diagnosis (years)	40.3 ± 11.6 (*N* = 66)	49.0 ± 9.2 (*N* = 9)	**0.025 ** ^3^
Age at disease onset (years)	28.4 ± 13.6 (*N* = 62)	37.0 ± 13.0 (*N*= 8)	0.116 ^3^
Disease form			**0.023 ** ^2^
Childhood/juvenile	25/63 (39.7%)	0/9 (0.0%)	
Adult/late	38/63 (60.3%)	9/9 (100.0%)	
Disease duration (years)	12.2 ± 11.6 (*N* = 63)	12.4 ± 11.6 (*N* = 8)	0.956 ^4^
Education (years)	11.1 ± 2.2 (*N* = 41)	13.3 ± 2.0 (*N* = 7)	**0.024 ** ^3^

*N*, number of available patients for a given variable; *n*, number of patients with the given characteristic. ^1^ *p*-values for comparison between CTG pure and variant repeat groups; ^2^ Fisher’s exact test; ^3^ Welch’s *t*-test; ^4^ Mann–Whitney U test. Continuous variables are presented as mean ± standard deviation, and categorical variables as *n*/*N* (%). Bold values indicate statistical significance at *p* < 0.05.

**Table 2:** Neuromuscular characteristics in consecutive patients with myotonic dystrophy type 1 with pure and variant repeats at the age at diagnosis.

Variable	Pure Repeats	Variant Repeats	*p*-Value ^1^
MIRS score	3.27 ± 0.77 (*N* = 63)	3.50 ± 1.07 (*N* = 8)	0.430 ^2^
MIRS = 1	0/63 (0.0%)	0/8 (0.0%)	
MIRS = 2	11/63 (17.5%)	2/8 (25.0%)	
MIRS = 3	25/63 (39.7%)	1/8 (12.5%)	
MIRS = 4	26/63 (41.3%)	4/8 (50.0%)	
MIRS = 5	1/63 (1.6%)	1/8 (12.5%)	
MIRS category			0.454 ^3^
Mild (1–3)	36/63 (57.1%)	3/8 (37.5%)	
Severe (4–5)	27/63 (42.9%)	5/8 (62.5%)	
Total MRC score (0–20)	16.18 ± 1.84 (*N* = 61)	15.63 ± 2.83 (*N* = 8)	0.582 ^2^
Upper extremity–proximal	4.66 ± 0.48	4.50 ± 0.76	0.692 ^2^
Upper extremity–distal	3.57 ± 0.69	3.25 ± 0.89	0.189 ^2^
Lower extremity–proximal	4.69 ± 0.50	4.25 ± 0.71	**0.049 ** ^2^
Lower extremity–distal	3.26 ± 0.89	3.63 ± 1.06	0.156 ^2^
Ptosis	56/66 (84.8%)	6/7 (85.7%)	1.000 ^3^
Facial muscle weakness	63/66 (95.5%)	7/8 (87.5%)	0.374 ^3^
Nasal speech	51/64 (79.7%)	4/8 (50.0%)	0.083 ^3^
Dysarthria (self-reported)	35/64 (54.7%)	4/8 (50.0%)	1.000 ^3^
Dysphagia (self-reported)	27/64 (42.2%)	4/8 (50.0%)	0.721 ^3^
Hand locking (self-reported)	55/65 (84.6%)	9/9 (100.0%)	0.347 ^3^
Grip myotonia at testing	48/51 (94.1%)	9/9 (100.0%)	1.000 ^3^
Percussion myotonia	48/51 (94.1%)	6/6 (100.0%)	1.000 ^3^
Masseter myotonia	24/49 (49.0%)	5/6 (83.3%)	0.197 ^3^
Tongue myotonia	20/37 (54.1%)	5/8 (62.5%)	0.716 ^3^
Masticatory muscle hypotrophy and weakness	54/63 (85.7%)	6/7 (85.7%)	1.000 ^3^
Sternocleidomastoid muscle hypotrophy and weakness	62/65 (95.4%)	8/9 (88.9%)	0.412 ^3^
Trapezius muscle hypotrophy and weakness	11/57 (19.3%)	2/6 (33.3%)	0.595 ^3^
Gait difficulty (self-reported)	37/64 (57.8%)	6/8 (75.0%)	0.470 ^3^
Ambulation impairment	3/64 (4.7%)	2/9 (22.2%)	0.152 ^3^

^1^ *p*-values for comparison between pure and variant repeat groups; ^2^ Mann–Whitney U test; ^3^ Fisher’s exact test; Continuous variables are presented as mean ± standard deviation, and categorical variables as *n*/*N* (%), where *n* is the number of patients with the characteristic and *N* is the number of available patients for that variable. MIRS, Muscular Impairment Rating Scale; MRC, Medical Research Council. Muscle strength for upper and lower extremities were analyzed separately for proximal and distal muscle groups. Total MRC score was obtained by summing the MRC score for the weakest muscle per group. Bold values indicate statistical significance at *p* < 0.05.

**Table 3:** Multisystem involvement in consecutive patients with myotonic dystrophy type 1 with pure and variant repeats at the age at diagnosis.

Variable	Pure Repeats	Variant Repeats	*p*-Value ^1^
Skeletal features			
Kyphosis	2/39 (5.1%)	0/6 (0.0%)	1.000
Scoliosis	1/37 (2.7%)	0/6 (0.0%)	1.000
Pronounced lumbar lordosis	1/37 (2.7%)	1/6 (16.7%)	0.262
High-arched palate	48/61 (78.7%)	5/7 (71.4%)	0.645
Electrophysiological findings			
Electromyography–myotonia	53/57 (93.0%)	8/8 (100.0%)	1.000
Electromyography–myopathy	42/57 (73.7%)	7/8 (87.5%)	0.650
Electroneurography–polyneuropathy	19/46 (41.3%)	0/5 (0.0%)	0.143
Respiratory involvement			
Reduced forced vital capacity	9/36 (25.0%)	3/5 (60.0%)	0.141
Non-invasive ventilation use	2/64 (3.1%)	1/8 (12.5%)	0.309
Cardiac manifestations			
Cardiac symptoms (self-reported)	10/58 (17.2%)	3/9 (33.3%)	0.361
Severe ECG abnormalities	8/44 (18.2%)	2/8 (25.0%)	1.000
Pacemaker implantation	2/64 (3.1%)	1/8 (12.5%)	0.309
Ophthalmological features			
Cataracts	28/57 (49.1%)	6/7 (85.7%)	0.109
Endocrine features			
Infertility	4/34 (11.8%)	1/6 (16.7%)	1.000
Glucose metabolism abnormalities	11/42 (26.2%)	1/6 (16.7%)	1.000

^1^ *p*-values for comparison between pure and variant repeat groups; All values are presented as *n*/*N* (%) where *n* is the number of patients with the characteristic and *N* is the number of available patients for that variable. All comparisons between groups were performed using Fisher’s exact test.

**Table 4:** Metabolic and laboratory parameters in consecutive patients with myotonic dystrophy type 1 with pure and variant repeats at the age at diagnosis.

Variable	Pure Repeats	Variant Repeats	*p*-Value ^1^
Creatine kinase (U/L)	227.2 ± 164.2 (*N* = 39)	326.7 ± 157.0 (*N* = 7)	0.071 ^2^
Elevated total cholesterol	16/38 (42.1%)	2/6 (33.3%)	1.000 ^3^
Elevated triglycerides	19/40 (47.5%)	1/5 (20.0%)	0.363 ^3^

^1^ *p*-values for comparison between pure and variant repeat groups; ^2^ Mann–Whitney U test; ^3^ Fisher’s exact test; Continuous variable is presented as mean ± standard deviation, and categorical variables as *n*/*N* (%), where *n* is the number of patients with the characteristic and *N* is the number of available patients for that variable. Elevated creatine kinase was defined as serum CK ≥ 200 U/L. Elevated total cholesterol was defined as total cholesterol ≥ 5.2 mmol/L. Elevated triglycerides were defined as triglycerides ≥ 1.7 mmol/L.

## Data Availability

The datasets analyzed during the current study, including patient clinical and genetic data, are not publicly available due to privacy and ethical concerns. Anonymized data may be made available from the corresponding author upon request.

## Abbreviations

The following abbreviations are used in this manuscript:

DM1	Myotonic dystrophy type 1
DM2	Myotonic dystrophy type 2
*DMPK*	Dystrophia myotonica protein kinase
*CNBP*	CCHC-type zinc finger nucleic acid binding protein
RP-PCR	Repeat-primed polymerase chain reaction
MIRS	Muscular Impairment Rating Scale
MRC	Medical Research Council
ECG	Electrocardiogram
CK	Creatine kinase
FVC	Forced vital capacity
AV	Atrioventricular
